# Spatial distribution and risk factors of adverse treatment outcomes of tuberculosis in Guizhou, China, 2013–2018

**DOI:** 10.1038/s41598-021-86994-6

**Published:** 2021-04-08

**Authors:** Jian Zhou, Xiaoxue Ma, Juan Tian, Feng Hong, Jinlan Li

**Affiliations:** 1 School of Public Health, The Key Laboratory of Environmental Pollution Monitoring and Disease Control, Ministry of Education, Guizhou Medical University, Guiyang, 550025 Guizhou China; 2 The First People’s Hospital of Guiyang, Guiyang, 550001 Guizhou China; 3 Guizhou Provincial Center for Disease Control and Prevention, The Institute of Tuberculosis Control and Prevention, Guiyang, 550001 Guizhou China

**Keywords:** Diseases, Health care, Health occupations, Risk factors

## Abstract

The incidence of Tuberculosis (TB) in Guizhou province has ranked to be the top four among the 31 China provinces. The spatial distribution and influencing factors of adverse outcomes of TB in Guizhou are unclear. In our study, the cases information of TB in Guizhou province from 2013 to 2018 was collected, we analyzed the spatial distribution and clusters of five adverse outcomes of TB with ArcMap10.2 software, used logistics regression analysis to assessed risk factors and used Chi-square analysis to analyze variation trend of the five adverse outcomes. A total of 237, 806 cases information of TB were collected. The proportion of adverse outcomes in TB patients was 6.18%, among which adverse reactions accounted for 1.05%, lost to follow-up accounted for 1.44%, treatment failed accounted for 1.15%, died accounted for 2.31%, switch to MDR accounted for 0.24%. The component ratio of adverse outcomes showed an upward trend (*P* < 0.05).Regional clustering existed in each of adverse outcomes (*P* < 0.05). There were high-risk minorities, gender, age, occupation, type of diagnosis, Therapeutic category existed in adverse outcomes of TB. Miao and Dong had a higher risk in adverse reaction of TB compared with Han. Women had a higher risk in adverse reactions than men, and a lower risk of lost to follow-up, failed, and died. Retreated patients had a higher risk of adverse outcomes. Timely monitoring and active intervention should be carried out for some high-risk areas and groups, including middle-aged and elderly patients, rural patients, floating patients, severe patients and retreated patients during the process of patient diagnosis and treatment.

## Introduction

**Figure 1:**
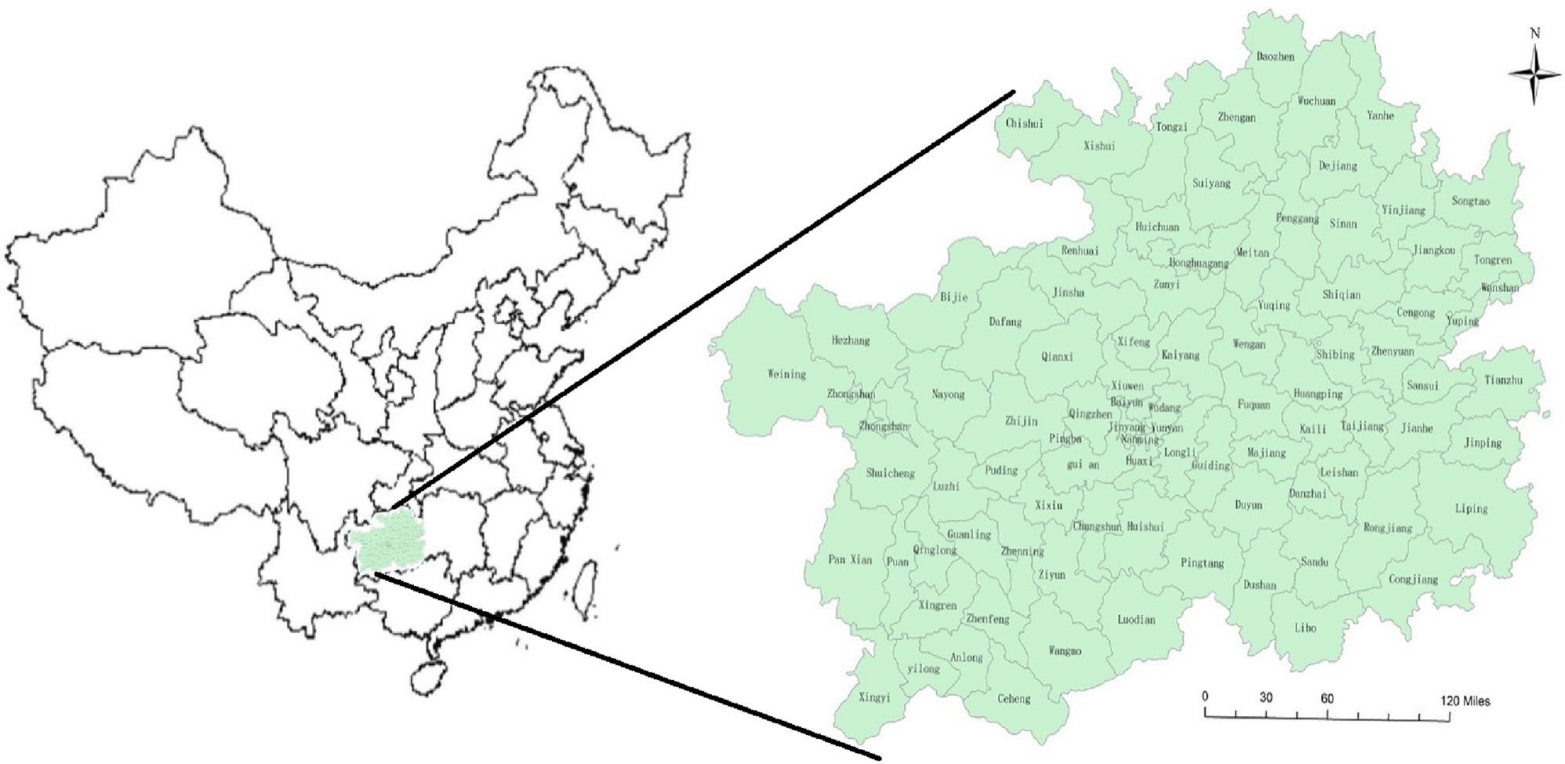
Location of Guizhou Province, China (ArcGis 10.2 software).

Tuberculosis (TB) is a chronic respiratory infectious disease caused by mycobacterium tuberculosis, which is one of the top ten causes of death worldwide, it is a serious infectious disease in some developing countries. China is one of 22 high TB burden countries in the world^1^. Male, farmers and middle-aged people are the main groups of TB patients. The incidence of TB in western China is higher than in central and eastern^2^. In 2018, China ranked the fourth in the world with incidence of 59/100,000^3^. Guizhou province is located in the west of China with 88 counties and 49 ethnic minorities (Fig. 1). From 2013 to 2018, more than 40,000 TB cases were reported in Guizhou province every year^4^, the reported incidence of TB in 2019 was 102/100,000. In addition, the treatment of TB is not entirely free in China, many patients' families were not rich, who need to pay about 22% of the total family income during the treatment process^5,6^. It will bring greater mental harm and economic burden to the patients and their families if these patients have a bad outcome of TB^7,8^.

It has been widely seen to analyze the epidemic characteristics of TB with spatial and temporal method^9–12^. Spatial–temporal analysis extends the analysis of epidemics in spatial epidemiology^13,14^, which can predict the spatial–temporal trends and correlations of diseases, explore spatial distribution models in spatial epidemiology^15^, objectively present the distribution characteristics of diseases with quantitative methods, and describe the clustering characteristics between time and space with scanning statistics^16,17^. But this method has rarely been used in studies of adverse outcomes of TB^18–21^, previous studies did not analyze every adverse outcomes of TB at the same time. However, it was rare to analyze adverse outcomes of TB with spatial distribution analysis^22–27^. Meanwhile, the spatial distribution and risk factors of adverse outcomes of TB in Guizhou are unclear. Our research’s purpose was to analyze the time and space characteristics of five adverse outcomes of TB, and assessed the risk factors and variation trend of the adverse outcomes of TB, so as to provide the evidence for timely monitoring and active intervention of the high-risk areas and groups in Guizhou province.

## Materials and methods

### Date source

Our data of TB cases came from *China information system of disease prevention and control*, the data was downloaded according to the conditions of onset date, current address, audited status. Through this system, the medical record information of all registered and managed TB patients in 88 counties of Guizhou province from 2013 to 2018 was obtained, among which the cases with unknown address, changed diagnosis, no treatment outcome or suspected diagnosis were excluded. The population data came from *the Statistics Bureau of Guizhou Province* in the same period.

### Indicators and definitions

The outcomes of TB treatment include cured, treatment completed, adverse reactions, lost to follow-up, treatment failed, died, and switch to multi-drug resistance (switch to MDR), these indicators are defined in accordance with World Health Organization (WHO) standards^28^, adverse reactions are included in the treatment failure in this standard, but which are used as a separate evaluation index in our study. In addition, we also used switch to MDR as one of the evaluation indicators of adverse outcomes. So there are five adverse outcomes of TB in our study, including adverse reactions, lost to follow-up, treatment failed, died and switch to MDR. The outcomes of TB treatment are defined as follows:

*Cured* Treatment completed as recommended by the national policy without evidence of failure and three or more consecutive cultures taken at least 30 days apart are negative after the intensive phase^28^.

*Treatment completed* Treatment completed as recommended by the national policy without evidence of failure but no record that three or more consecutive cultures taken at least 30 days apart are negative after the intensive phase^28^.

*Treatment failed* Treatment terminated or need for permanent regimen change of at least two anti-TB drugs because of: Lack of conversion by the end of the intensive phase; or bacteriological reversion in the continuation phase after conversion to negative; or evidence of additional acquired resistance to fluoroquinolones or second-line injectable drugs^28^.

*Died* A patient who dies for any reason during the course of treatment^28^.

*Lost to follow-up* A patient whose treatment was interrupted for two consecutive months or more^28^.

*Adverse reactions* A harmful and/or undesirable reaction caused by a prescribed drug in a treated patient when used in the correct manner and at the correct dosage^28^.

*Switch to MDR* An ordinary TB patient receiving treatment develops resistance to one or more anti-TB drugs after a period of treatment.

Component ratio (CR) of adverse outcomes of TB refers to the proportion of the number of TB patients with adverse outcome accounting for the total number of TB patients in this year, including the CR of adverse reaction, the CR of lost to follow-up, the CR of treatment failed, the CR of died and CR of switch to MDR, which are used to describe the variation trend of adverse outcomes of TB.

Rate of adverse outcomes refers to the proportion of the number of TB patients with adverse outcome in the total number of TB patients in a region, we take 1/1000 as the proportion unit, including rate of adverse reaction, rate of lost to follow-up, rate of treatment failed, rate of died and rate of switch to MDR, which are used to describe the spatial distribution of adverse outcome of TB.

In the analysis of risk factors, the factors included gender, age, nationality, occupation, local registered residence, source of patients, clinical classification, diagnostic results, severe cases, and therapeutic category.

### Spatial analysis

According to the cases of current address, we got the case's national standard area code's information, the spatial location of TB patients would be associated with vectorized electronic maps of Guizhou, the prevalence of adverse outcomes of TB in counties (districts) would be established in the form of geographic information, and ArcMap10.2 software was used to calculate the local indicators of spatial association (LISA), among which there are four types of cluster diagrams, including High–High (HH), Low–Low (LL), High–Low (HL) and Low–High (LH). The HH indicates that the rate of adverse outcomes in the region is high and the rate of adverse outcomes in neighboring region is still high. The LL indicates that the rate of adverse outcomes in the region is low and the rate of adverse outcomes in neighboring region is still low. The HL indicates that the rate of adverse outcomes in the region is high and the rate of adverse outcomes in neighboring region is low. The LH indicates that the rate of adverse outcomes in the region is low and the rate of adverse outcomes in neighboring region is high.

### Analysis of risk factors

In the study of the risk factors, the factors included gender, age, nationality, occupation, local registered residence, source of patients, clinical classification, diagnostic results, severe cases, and therapeutic category. We first conducted univariate logistics regression analysis for each adverse outcome. When univariate logistics analysis was statistically significant (*P* < 0.05), the factor would be subjected to multivariable logistics regression analysis, and the statistical software was SPSS22.0.

## Results

**Figure 2:**
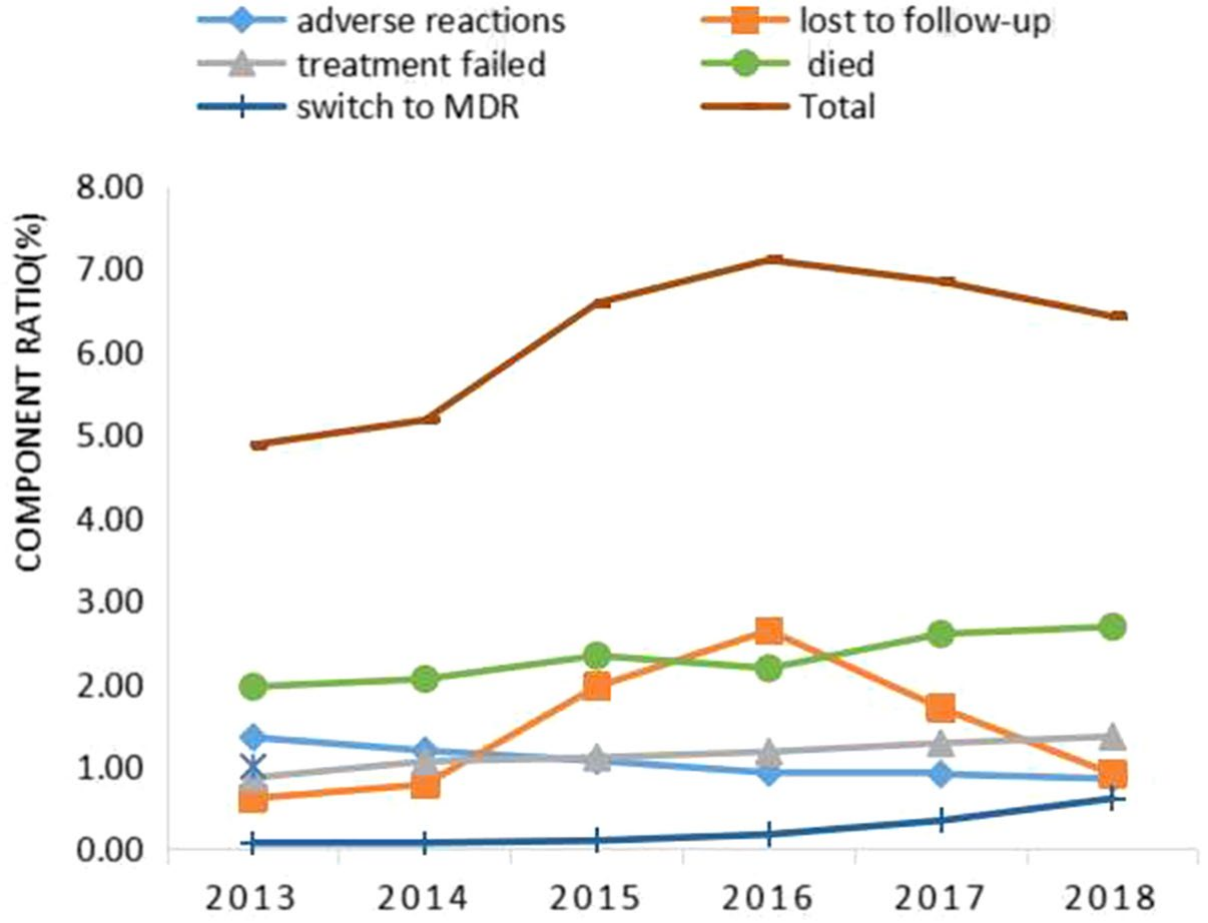
Variation trends (*p* < 0.05) of the component ratio (CR) of adverse outcomes in TB Patients (%), Guizhou, China, 2013–2018.

We obtained a total of medical record of 237,806 cases according to the requirements of data in our study, including 40,381 cases in 2013, 37,838 in 2014, 39,266 in 2015, 39,611 in 2016, 39,622 in 2017, and 41,088 in 2018.The proportion of adverse outcomes in TB patients was 6.18%, among which adverse reactions accounted for 1.05%, lost to follow-up accounted for 1.44%, treatment failed accounted for 1.15%, died accounted for 2.31%, and switch to MDR accounted for 0.24%. The CR of total adverse outcomes showed an upward trend (*P* < 0.05), among which except the CR of adverse reactions showed a downward trend (*P* < 0.05), and the CR of lost to follow-up, treatment failed, died and switch to MDR showed an upward trend (*P* < 0.05) (Fig. 2).

### Spatial distribution and clusters

**Figure 3:**
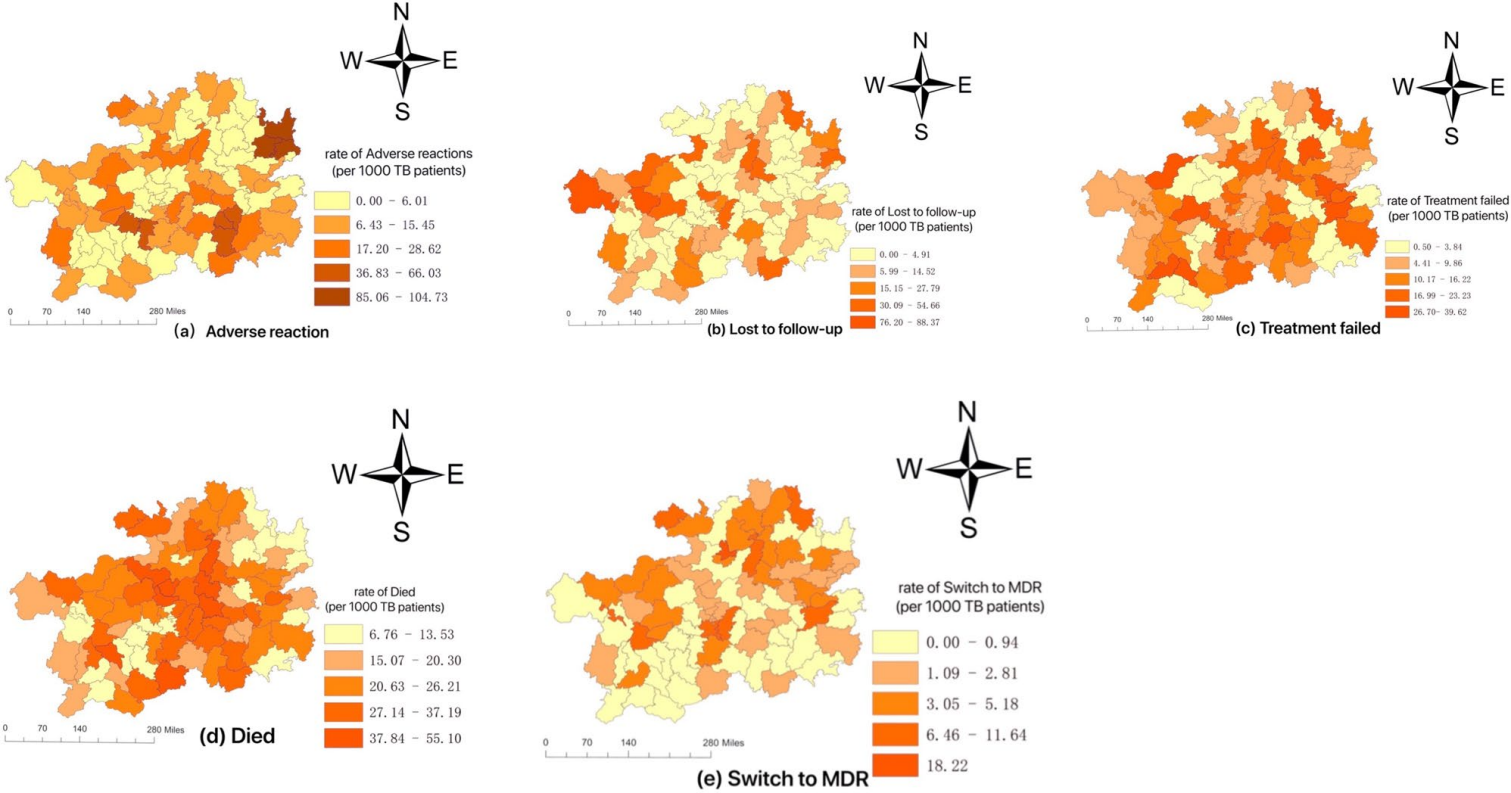
The spatial distribution of adverse outcomes of TB in Guizhou during 2013–2018. (**a**) Geographic distribution of rate of adverse reaction; (**b**) Geographic distribution of rate of lost to follow-up; (**c**) Geographic distribution of rate of treatment failed; (**d**) Geographic distribution of rate of died; (**e**) Geographic distribution of rate of switch to MDR.

**Figure 4:**
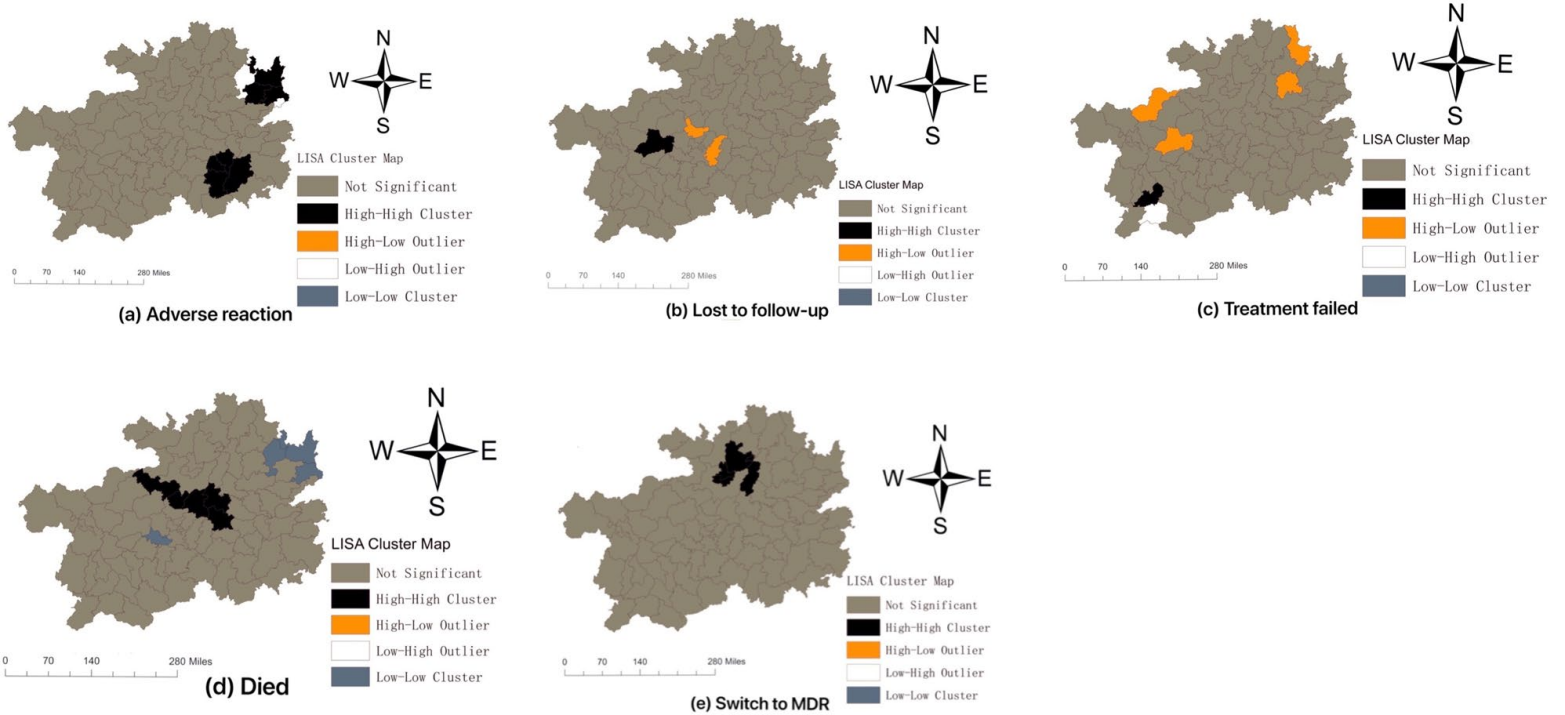
Maps of the local indicators of spatial association (LISA) of adverse outcomes of TB in Guizhou during 2013–2018. (**a**) Spatial clusters of rate of adverse reaction; (**b**) Spatial clusters of rate of lost to follow-up; (**c**) Spatial clusters of rate of treatment failed; (**d**) Spatial clusters of rate of died; (**e**) Spatial clusters of rate of switch to MDR.

The spatial distribution of adverse outcomes of TB in Guizhou were irregular, and the five adverse outcomes of TB showed their own distribution characteristics (Fig. 3). The five adverse outcomes showed their own characteristics of spatial clusters (Fig. 4).

There were two clusters in adverse reactions of TB in Guizhou, which contained Tongren City (including Wanshan, Dejiang, Yuping, Bijiang County) and Qiandongnan city (including Rongjiang, Leishan, Danzhai County) (Fig. 4a).

There was a cluster in lost to follow-up of TB in Guizhou (Fig. 4b). Zhijin County (Bijie City) was the region with statistical significance (*P* < 0.05) in HH (Fig. 4b), and there were two regions (including Longli and Xiuwen County) with statistical significance (*P* < 0.05) in HL (Fig. 4b).

Xingren County was a region with statistical significance (*P* < 0.05) in HH about treatment failed of TB in Guizhou (Fig. 4c), there were four regions with statistical significance (*P* < 0.05) in HL, including Qixingguan, Zhijin, Songtao and Yinjiang County (Fig. 4c). Anlong County was the region with statistical significance (*P* < 0.05) in LH (Fig. 4c).

There was a cluster in died of TB in Guizhou (Fig. 4d). There were five regions with statistical significance (*P* < 0.05) in HH, including Jinsha, Xifeng, Kaiyang, Fuquan and Wengan County, there were four regions with statistical significance (*P* < 0.05) in LL, including Dejiang, Wanshan, Bijiang and Pingba County (Fig. 4d).

There was a cluster in switch to MDR of TB in Guizhou (Fig. 4e). There were four regions with statistical significance (*P* < 0.05) in HH, including Suiyang, Huichuan, Honghuagang and Meitan County, these four areas were concentrated in Zunyi City (Fig. 4e).

### Risk factors

In our study on risk factors of adverse reactions in TB patients, women had a higher risk compared with men. The higher the age, the higher the risk. Miao, Dong and Others had a higher risk compared with Han, Buyi and Tujia had a lower risk. Compared with peasant workers, the homemakers and unemployed persons, Retired persons and cadres and staffs had a higher risk, while students had a lower risk. Compared with local patients, the non-local patients had a lower risk. Recommendation for symptoms had a higher risk compared to clinical consultation. Compared with positive etiology, the negative etiology and pure tuberculous pleurisy had a higher risk. Non-severe had a higher risk compared to severe. Re-treatment had a higher risk compared with initial treatment (Table 1).

**Table 1:** Results of risk factors analysis of adverse reactions in TB patients.

Variables	n	Rate of adverse reactions (per 1000 TB)	Univariable analysis		Multivariable analysis	
			OR(95%CI)	*P*-value	OR(95%CI)	*P*-value
**Gender**						
Males	1579	66.40	Referent		Referent	
Females	916	38.52	1.12 (1.04–1.22)	0.005	1.15 (1.06–1.25)	0.001
**Age**						
0~	126	5.30	Referent		Referent	
20~	529	22.25	1.63 (1.34–1.98)	< 0.001	1.49 (1.21–1.84)	< 0.001
40~	756	31.79	2.37 (1.96–2.86)	< 0.001	2.20 (1.79–2.71)	< 0.001
60~	1084	45.58	3.99 (3.32–4.84)	< 0.001	3.69 (3.01–4.52)	< 0.001
**Nationality**						
Han	1570	66.02	Referent		Referent	
Miao	372	15.64	1.30 (1.17–1.46)	< 0.001	1.38 (1.23–1.54)	< 0.001
Buyi	154	6.48	0.82 (0.70–0.97)	0.022	0.83 (0.70–0.98)	0.031
Yi	51	2.14	0.75 (0.57–1.00)	0.046	0.89 (0.67–1.18)	0.412
Dong	152	6.39	1.75 (1.48–2.07)	< 0.001	1.88 (1.59–2.23)	< 0.001
Tujia	71	2.99	0.61 (0.48–0.77)	< 0.001	0.59 (0.47–0.75)	< 0.001
Others	125	5.26	1.49 (1.24–1.79)	< 0.001	1.59 (1.32–1.91)	< 0.001
**Occupation**						
Peasant workers	2082	87.55	Referent		Referent	
Homemakers and unemployed persons	153	6.43	1.26 (1.07–1.49)	0.006	1.60 (1.35–1.90)	< 0.001
Students	91	3.83	0.43 (0.35–0.54)	< 0.001	0.73 (0.58–0.93)	0.009
Retired persons and cadres and staff	94	3.95	1.94 (1.57–2.38)	< 0.001	1.68 (1.36–2.07)	< 0.001
Others	75	3.15	0.72 (0.57–0.90)	0.005	0.91 (0.72–1.15)	0.420
**Local registered residence**						
Yes	2305	96.93	Referent		Referent	
No	190	7.99	0.74 (0.64–0.86)	< 0.001	0.78 (0.66–0.90)	0.001
**Source of patients**						
Clinical consultation	1134	47.69	Referent		Referent	
Referral	787	33.09	1.01 (0.92–1.11)	0.862	0.97 (0.89–1.07)	0.543
Following up	413	17.37	1.00 (0.90–1.12)	0.956	1.02 (0.91–1.15)	0.740
Recommended for symptoms	88	3.70	1.97(1.58–2.45)	< 0.001	2.17 (1.74–2.70)	0.001
Physical examinations	41	1.72	0.73 (0.53–0.99)	0.046	1.13 (0.82–1.55)	0.460
Others	32	1.35	1.08 (0.76–1.54)	0.677	1.16 (0.82–1.66)	0.405
**Clinical classification**						
Type I	0	0.00	Referent			
Type II	51	2.14	0.00	0.996		
Type III	2375	99.87	0.83 (0.39–1.75)	0.619		
Type IV	61	2.57	0.68 (0.34–1.36)	0.275		
Type V	8	0.34	0.66 (0.32–1.39)	0.274		
**Diagnosis**						
Etiological positivity	723	30.40	Referent		Referent	
Etiological negative	1709	71.87	1.14 (1.05–1.24)	0.003	1.27 (1.17–1.41)	< 0.001
Pure tuberculous pleurisy	55	2.31	1.41 (1.07–1.85)	0.015	1.81 (1.37–2.39)	< 0.001
Others	8	0.34	1.10 (0.54–2.21)	0.800	1.52 (0.75–3.07)	0.246
**Severe cases**						
Yes	296	12.45	Referent		Referent	
No	2199	92.47	1.20 (1.06–1.36)	0.003	1.23 (1.09–1.39)	0.001
**Therapeutic category**						
Initial treatment	2250	94.61	Referent		Referent	
Re-treated	245	10.30	1.62 (1.42–1.85)	< 0.001	1.65 (1.44–1.90)	< 0.001

In the study on risk factors of lost to follow up, women had a lower risk compared with men. The higher risk was 20 to 39 years compared with 0 to 19 years. Compared with Han, Yi had a higher risk, Buyi, Dong, Tujia and Others had a lower risk. Compared with peasant workers, the Retired persons and cadres and staffs, Others had a lower risk. Compared with local patients, the non-local had a higher risk. Referral and following up had a lower risk compared with clinical consultation. Compared with Type I, the Type III, Type IV and Type V had a higher risk. Pure tuberculous pleurisy and others had a higher risk compared with positive etiology. Non-severe had a lower risk compared to severe. Re-treatment had a higher risk compared with initial treatment (Table 2).

**Table 2:** Results of risk factors analysis of lost to follow up in TB patients.

Variables	n	Rate of Lost to follow up (per 1000 TB)	Univariable analysis		Multivariable analysis	
			OR (95%CI)	*P*-value	OR (95%CI)	*P*-value
**Gender**						
Males	2443	102.73	Referent		Referent	
Females	972	40.87	0.77 (0.71–0.83)	< 0.001	0.78 (0.72–0.84)	< 0.001
**Age**						
0~	382	16.06	Referent		Referent	
20~	1155	48.57	1.17 (1.04–1.32)	0.008	1.18 (1.04–1.35)	0.009
40~	1015	42.68	1.04 (0.93–1.17)	0.485	1.06 (0.93–1.20)	0.43
60~	863	36.29	1.04 (0.92–1.17)	0.578	1.13 (0.99–1.30)	0.076
**Nationality**						
Han	2420	101.76	Referent		Referent	
Miao	450	18.92	1.02 (0.92–1.13)	0.689	1.04 (0.94–1.15)	0.461
Buyi	163	6.85	0.56 (0.48–0.66)	< 0.001	0.57 (0.48–0.67)	< 0.001
Yi	200	8.41	1.95 (1.68–2.23)	< 0.001	1.82 (1.57–2.11)	< 0.001
Dong	53	2.23	0.39 (0.30–0.51)	< 0.001	0.39 (0.29–0.51)	< 0.001
Tujia	37	1.56	0.20 (0.15–0.28)	< 0.001	0.22 (0.16–0.30)	< 0.001
Others	92	3.87	0.71 (0.57–0.87)	0.001	0.71 (0.58–0.87)	0.001
**Occupation**						
Peasant workers	2787	117.20	Referent		Referent	
Homemakers and unemployed persons	192	8.07	1.18 (1.02–1.37)	0.026	1.12 (0.96–1.30)	0.148
Students	283	11.90	1.01 (0.90–1.15)	0.843	1.07 (0.93–1.23)	0.323
Retired persons and cadres and staff	36	1.51	0.54 (0.39–0.76)	< 0.001	0.56 (0.41–0.79)	0.001
Others	117	4.92	0.83 (0.69–1.01)	0.056	0.81 (0.67–0.98)	0.027
**Local registered residence**						
Yes	3067	128.97	Referent		Referent	
No	348	14.63	1.02 (0.91–1.14)	0.728	1.18 (1.05–1.32)	0.005
**Source of patients**						
Clinical consultation	1777	74.72	Referent		Referent	
Referral	1065	44.78	0.87 (0.81–0.94)	< 0.001	0.85 (0.79–0.92)	< 0.001
Following up	382	16.06	0.59 (0.53–0.66)	< 0.001	0.59 (0.52–0.66)	< 0.001
Recommended for symptoms	71	2.99	1.00 (0.79–1.27)	0.980	0.90 (0.71–1.15)	0.411
Physical examinations	73	3.07	0.83 (0.65–1.05)	0.112	0.87 (0.69–1.11)	0.268
Others	47	1.98	1.01 (0.75–1.35)	0.949	1.06 (0.79–1.42)	0.705
**Clinical classification**						
Type I	0	0.00	Referent		Referent	
Type II	62	2.61	0.00	0.996	0.00	0.996
Type III	3109	130.74	1.62 (0.65–4.05)	0.301	2.71 (1.06–6.93)	0.038
Type IV	239	10.05	1.43 (0.59–3.46)	0.424	2.74 (1.11–6.79)	0.029
Type V	5	0.21	4.30 (1.76–10.47)	0.001	3.93 (1.61–9.60)	0.003
**Diagnosis**						
Etiological positivity	1109	46.63	Referent		Referent	
Etiological negative	2083	87.59	0.90 (0.84–0.97)	0.007	1.06 (0.98–1.15)	0.142
Pure tuberculous pleurisy	205	8.62	3.53 (3.03–4.11)	< 0.001	3.28 (2.51–4.30)	< 0.001
Others	18	0.76	1.62 (1.01–2.59)	0.045	2.34 (1.45–3.77)	< 0.001
**Severe cases**						
Yes	652	27.42	Referent		Referent	
No	2763	116.19	0.68 (0.62–0.74)	< 0.001	0.67 (0.62–0.74)	< 0.001
**Therapeutic category**						
Initial treatment	2965	124.68	Referent		Referent	
Re-treated	450	18.92	2.29 (2.07–2.53)	< 0.001	2.17 (1.96–2.42)	< 0.001

In the study on risk factors of treatment failed, women had a lower risk compared with men. Over 20 years of age had a higher risk compared with 0 to 19 years. The differences between ethnic groups were not statistically significant. Compared with peasant workers, others had a lower risk. There was no statistically significant difference between regions. Compared with clinical consultation, Recommendation for symptoms had a higher risk, while referral and following-up had a lower risk. Compared with positive etiology, negative etiology, pure tuberculous pleurisy and others had a lower risk. Non-severe had lower risk compared to severe. Retreatment had a higher risk compared with initial treatment (Table 3).

**Table 3:** Results of risk factors analysis of treatment failed in TB patients.

Variables	n	Rate of treatment failed (per 1000 TB)	Univariable analysis		Multivariable analysis	
			OR (95%CI)	*P*-value	OR (95%CI)	*P*-value
**Gender**						
Males	2055	86.41	Referent		Referent	
Females	668	28.09	0.63 (0.57–0.68)	< 0.001	0.72 (0.66–0.78)	< 0.001
**Age**						
0~	148	6.22	Referent		Referent	
20~	643	27.04	1.68 (1.41–2.01)	< 0.001	1.34 (1.12–1.62)	0.003
40~	1140	47.94	3.05 (2.57–3.63)	< 0.001	2.17 (1.80–2.61)	< 0.001
60~	792	33.30	2.47 (2.07–2.95)	<0.001	1.89 (1.56–2.28)	< 0.001
**Nationality**						
Han	1789	75.23	Referent		Referent	
Miao	326	13.71	1.00 (0.89–1.13)	0.998	0.94 (0.83–1.06)	0.279
Buyi	239	10.05	1.13 (0.98–1.29)	0.089	1.08 (0.94–1.24)	0.289
Yi	77	3.24	1.95 (1.68–2.23)	0.993	0.99 (0.79–1.25)	0.946
Dong	98	4.12	1.00 (0.79–1.26)	0.862	0.83 (0.68–1.02)	0.083
Tujia	122	5.13	0.98 (0.80–1.21)	0.384	0.97 (0.81–1.17)	0.758
Others	72	3.03	0.92 (0.77–1.11)	0.016	0.80 (0.63–1.01)	0.058
**Occupation**						
Peasant workers	2340	98.40	Referent		Referent	
Homemakers and unemployed persons	137	5.76	1.00 (0.84–1.19)	0.977	1.11 (0.93–1.33)	0.237
Students	131	5.51	0.56 (0.47–0.66)	< 0.001	0.92 (0.76–1.11)	0.380
Retired persons and cadres and staff	43	1.81	0.78 (0.57–1.05)	0.104	0.77 (0.57–1.05)	0.059
Others	72	3.03	0.61 (0.48–0.77)	< 0.001	0.72 (0.57–0.91)	0.007
**Local registered residence**						
Yes	2489	104.67	Referent		Referent	
No	234	9.84	0.84 (0.74–0.97)	0.013	0.97 (0.84–1.11)	0.652
**Source of patients**						
Clinical consultation	1323	55.63	Referent		Referent	
Referral	836	35.15	0.92 (0.84–1.00)	0.051	0.87 (0.80–0.95)	0.002
Following up	384	16.15	0.80 (0.71–0.89)	< 0.001	0.86 (0.76–0.97)	0.011
Recommended for symptoms	96	4.04	1.84 (1.49–2.27)	< 0.001	1.49 (1.20–1.84)	< 0.001
Physical examinations	33	1.39	0.50 (0.35–0.71)	< 0.001	0.98 (0.69–1.39)	0.897
Others	51	2.14	1.48 (1.12–1.96)	0.006	1.29 (0.97–1.71)	0.086
**Clinical classification**						
Type I	0	0.00	Referent			
Type II	25	1.05	10,145,695.39 (0.00)	0.996		
Type III	2673	112.40	19,244,084.80 (0.00)	0.996		
Type IV	25	1.05	6,785,381.10 (0.00)	0.996		
Type V	0	0.00	1.00 (0.00)	1.000		
**Diagnosis**						
Etiological positivity	1758	73.93	Referent		Referent	
Etiological negative	950	39.95	0.26 (0.24–0.28)	< 0.001	0.31 (0.29–0.34)	< 0.001
Pure tuberculous pleurisy	11	0.46	0.11 (0.06–0.20)	< 0.001	0.17 (0.09–0.30)	< 0.001
Others	4	0.17	0.22 (0.08–0.59)	0.003	0.34 (0.13–0.91)	0.032
**Severe cases**						
Yes	782	32.88	Referent		Referent	
No	1941	81.62	0.40 (0.36–0.43)	< 0.001	0.48 (0.44–0.52)	< 0.001
**Therapeutic category**						
Initial treatment	2224	93.52	Referent		Referent	
Re-treated	499	20.98	3.40 (3.08–3.75)	< 0.001	1.87 (1.69–2.07)	< 0.001

In the study on risk factors of died, women had a lower risk compared with men. The risk increases with age. Compared with Han, Yi had a higher risk, Dong and Tujia had a lower risk. Students had a higher risk compared with peasant workers, while homemakers and unemployed persons, Retired persons and cadres and staffs and others had a lower risk. Compared with local patients, the non-local had a lower risk. Compared with clinical consultation, referral had a higher risk, while physical examination had a lower risk. Compared with positive etiology, negative etiology had a lower risk. Non-severe had a lower risk compared to severe. Retreatment had a higher risk compared with initial treatment (Table 4).

**Table 4:** Results of risk factors analysis of died in TB patients.

Variables	n	Rate of died (per 1000 TB)	Univariable analysis		Multivariable analysis	
			OR (95%CI)	*P*-value	OR (95%CI)	*P*-value
**Gender**						
Males	3989	167.74	Referent		Referent	
Females	1503	63.20	0.72 (0.68–0.77)	< 0.001	0.72 (0.68–0.76)	< 0.001
**Age**						
0~	77	3.24	Referent		Referent	
20~	514	21.61	2.59 (2.04–3.29)	< 0.001	2.46 (1.92–3.15)	< 0.001
40~	1496	62.91	7.76 (6.17–9.76)	< 0.001	6.89 (5.48–8.75)	< 0.001
60~	3405	143.18	21.36 (17.04–26.79)	< 0.001	19.67 (15.52–24.93)	< 0.001
**Nationality**						
Han	3739	157.23	Referent		Referent	
Miao	650	27.33	0.95 (0.88–1.04)	0.264	0.96 (0.88–1.05)	0.343
Buyi	443	18.63	1.00 (0.90–1.10)	0.940	0.98 (0.88–1.08)	0.630
Yi	146	6.14	0.90 (0.77–1.07)	0.238	1.23 (1.03–1.46)	0.017
Dong	170	7.15	0.81 (0.70–0.95)	0.008	0.74 (0.63–0.87)	< 0.001
Tujia	162	6.81	0.58 (0.50–0.68)	< 0.001	0.52 (0.45–0.62)	< 0.001
Others	182	7.65	0.91 (0.78–1.05)	0.192	1.00 (0.86–1.16)	0.968
**Occupation**						
Peasant workers	4911	206.51	Referent		Referent	
Homemakers and unemployed persons	157	6.60	0.54 (0.46–0.64)	< 0.001	0.83 (0.71–0.98)	0.028
Students	180	7.57	0.36 (0.31–0.42)	< 0.001	1.19 (1.01–1.39)	0.036
Retired persons and cadres and staff	138	5.80	1.20 (1.01–1.42)	0.039	0.82 (0.69–0.98)	0.026
Others	106	4.46	0.42 (0.35–0.51)	< 0.001	0.68 (0.56–0.83)	< 0.001
**Local registered residence**						
Yes	5127	215.60	Referent		Referent	
No	365	15.35	0.63 (0.57–0.71)	< 0.001	0.79 (0.71–0.89)	< 0.001
**Source of patients**						
Clinical consultation	2299	96.68	Referent		Referent	
Referral	2195	92.30	1.40 (1.32–1.48)	< 0.001	1.22 (1.15–1.30)	< 0.001
Following up	843	35.45	1.01 (0.93–1.09)	0.803	0.99 (0.91–1.07)	0.731
Recommended for symptoms	90	3.78	0.98 (0.79–1.22)	0.870	0.95 (0.77–1.18)	0.666
Physical examinations	9	0.38	0.08 (0.04–0.15)	< 0.001	0.22 (0.11–0.42)	< 0.001
Others	56	2.35	0.93 (0.71–1.21)	0.586	0.97 (0.74–1.28)	0.851
**Clinical classification**						
Type I	0	0.00	Referent			
Type II	242	10.18	107,473,172.80 (0.00)	0.996		
Type III	5110	214.88	38,482,807.67 (0.00)	0.996		
Type IV	134	5.63	38,332,749.21 (0.00)	0.996		
Type V	6	0.25	19,480,213.23 (0.00)	0.966		
**Diagnosis**						
Etiological positivity	2513	105.67	Referent		Referent	
Etiological negative	2878	121.02	0.54 (0.51–0.57)	< 0.001	0.62 (0.59–0.65)	< 0.001
Pure tuberculous pleurisy	88	3.70	0.64 (0.51–0.79)	< 0.001	0.91 (0.73–1.13)	0.391
Others	13	0.55	0.50 (0.29–0.87)	0.014	0.97 (0.55–1.69)	0.902
**Severe cases**						
Yes	1089	45.79	Referent		Referent	
No	4403	185.15	0.65 (0.60–0.69)	< 0.001	0.72 (0.67–0.77)	< 0.001
**Therapeutic category**						
Initial treatment	4900	206.05	Referent		Referent	
Re-treated	592	24.89	1.82 (1.67–1.99)	< 0.001	1.23 (1.12–1.34)	< 0.001

In the study on the risk factors of switch to MDR, there was no difference between the genders. The risk factor is 40 to 59 years compared with 0 to 19 years. Miao and Buyi had a lower risk compared with Han. Homemakers and unemployed, retired persons and cadres and staffs had a higher risk compared with peasant workers. Compared with local patients, the non-local had a higher risk. Compared with clinical consultation, referral and others had a higher risk, following-up had a lower risk. Compared with positive etiology, negative etiology had a lower risk. Non-severe had lower risk compared to severe. Retreatment had a higher risk compared with initial treatment (Table 5).

**Table 5:** Results of risk factors analysis of switch to MDR in TB patients.

Variables	n	Rate of switch to MDR (per 1000 TB)	Univariable analysis		Multivariable analysis	
			OR (95%CI)	*P*-value	OR (95%CI)	*P*-value
**Gender**						
Males	419	17.62	Referent		Referent	
Females	150	6.31	0.69 (0.57–0.83)	< 0.001	0.90 (0.75–1.09)	0.294
**Age**						
0~	29	1.22	Referent		Referent	
20~	179	7.53	2.39 (1.61–3.53)	< 0.001	1.39 (0.92–2.11)	0.116
40~	234	9.84	3.17 (2.16–4.67)	< 0.001	1.49 (0.98–2.25)	0.06
60~	127	5.34	2.01 (1.34–3.01)	0.001	1.02 (0.66–1.57)	0.938
**Nationality**						
Han	424	17.83	Referent		Referent	
Miao	53	2.23	0.69 (0.52–0.91)	0.01	0.650.49–0.87)	0.004
Buyi	16	0.67	0.32 (0.19–0.52)	< 0.001	0.32 (0.19–0.53)	< 0.001
Yi	19	0.80	1.04 (0.66–1.65)	0.867	0.94 (0.59–1.50)	0.801
Dong	15	0.63	0.63 (0.38–1.06)	0.083	0.65 (0.39–1.09)	0.104
Tujia	18	0.76	0.57 (0.36–0.92)	0.021	0.78 (0.48–1.26)	0.307
Others	24	1.01	1.06 (0.70–1.59)	0.800	1.16 (0.77–1.76)	0.481
**Occupation**						
Peasant workers	436	18.33	Referent		Referent	
Homemakers and unemployed persons	57	2.40	2.25 (1.70–2.96)	< 0.001	1.85 (1.38–2.47)	< 0.001
Students	32	1.35	0.73 (0.51–1.05)	0.088	1.12 (0.76–1.64)	0.570
Retired persons and cadres and staff	15	0.63	1.46 (0.87–2.45)	0.149	1.71 (1.01–2.90)	0.044
Others	29	1.22	1.33 (0.91–1.93)	1.326	1.40 (0.95–2.06)	0.087
**Local registered residence**						
Yes	471	19.81	Referent		Referent	
No	98	4.12	1.87 (1.51–2.33)	< 0.001	2.38 (1.88–3.01)	< 0.001
**Source of patients**						
Clinical consultation	254	10.68	Referent		Referent	
Referral	223	9.38	1.28 (1.07–1.53)	0.008	1.43 (1.19–1.72)	< 0.001
Following up	56	2.35	0.61 (0.45–0.81)	0.001	0.62 (0.46–0.83)	0.002
Recommended for symptoms	12	0.50	1.19 (0.66–2.12)	0.563	0.73 (0.40–1.32)	0.292
Physical examinations	5	0.21	0.40 (0.16–0.96)	0.041	1.00 (0.41–2.46)	0.998
Others	19	0.80	2.87 (1.80–4.58)	< 0.001	1.62 (1.00–2.62)	0.049
**Clinical classification**						
Type I	0	0.00	Referent			
Type II	3	0.13	1,210,203.72.39 (0.00)	0.996		
Type III	566	23.80	4,035,016.57 (0.00)	0.996		
Type IV	0	0.00	1.00 (0.00)	1.000		
Type V	0	0.00	1.00 (0.00)	1.000		
**Diagnosis**						
Etiological positivity	520	21.87	Referent		Referent	
Etiological negative	49	2.06	0.05 (0.03–0.06)	< 0.001	0.08 (0.06–0.11)	< 0.001
Pure tuberculous pleurisy	0	0.00	0.00 (0.00)	0.979	0.00 (0.00)	0.980
Others	0	0.00	0.00 (0.00)	0.991	0.00 (0.00)	0.991
**Severe cases**						
Yes	199	8.37	Referent		Referent	
No	370	15.56	0.30 (0.25–0.36)	< 0.001	0.42 (0.35–0.50)	< 0.001
**Therapeutic category**						
Initial treatment	301	12.66	Referent		Referent	
re-treated	268	11.27	16.95 (14.36–19.99)	< 0.001	8.14 (6.84–9.69)	< 0.001

## Discussions

We studied the variation trend, spatial distribution, and risk factor analysis of the five adverse outcomes of TB in Guizhou. Guizhou is a typical karst landscape with a humid and rainy climate. Compared with the eastern and central regions, it is underdeveloped in terms of economy, transportation, culture and education level. In addition, the population of ethnic minorities in Guizhou accounts for a large proportion with 49 ethnic minorities. Because the lifestyle, living environment of many ethnic minorities are different from others, what's more, we don't know if there are some genetic differences between them that might affect the outcome, therefore, ethnic minorities are also included in our study as an important risk factor.

Among the five adverse outcomes of TB, except that the CR of adverse reactions showed a downward trend (*P* < 0.05), and the CR of lost to follow-up, treatment failed, died and switch to MDR showed an upward trend (Fig. 1), which indicated that the adverse outcomes of TB in Guizhou were not satisfying and even worrying. The five adverse outcomes showed characteristics of spatial cluster, but the regions of spatial cluster were different. We can draw some conclusions from LISA diagram, the high-risk areas for adverse reaction of TB in Guizhou were mainly concentrated in Tongren city and Qiandongnan city (Fig. 3a). The spatial clustering of TB patients' loss of follow-up, treatment failure, and death were different, but there were also relevant and common evidences that revealed that the medical conditions in these areas and the living conditions of patients may need to be improved, because loss of follow-up, treatment failure, and death in TB patients are strongly related to patient compliance, living environment, family economic conditions, humanistic care and local medical conditions.The high-risk area for switch to MDR was mainly concentrated in Zunyi city (Fig. 3e). Compared with the regional ranking of TB incidence in Guizhou from 2013 to 2018^4^, it shows that the regions with high rate of adverse outcomes of TB in Guizhou were not only in those with high incidence, for example, Guiyang city had a low incidence of TB but high risk in died. It should be noticed by the relevant authorities.

Some studies have shown that poor economic conditions, poor medical services condition^18^, with diabetes^29^, old age and smoking will increase the risk of adverse reactions^30^, our study shows that women are more likely to have adverse reactions than men, Miao and Dong have a higher risk, while Buyi and Tujia have a lower risk. In addition, recommendation for symptoms, etiological negative, pure tuberculous pleurisy, middle-aged and elderly, retired persons and cadres and staffs, severe, re-treatment patients have a higher risk (Table 1).

Existing study^31^ have shown that the lost to follow-up of TB patients are significantly correlated with occupation, smoking, drinking, marital status, socioeconomic status, the study also found that although the drugs are provided freely, but there are many unfavorable factors for male patients, for example, low socioeconomic status, household debt and loss of income due to work and so on, which lead to them cannot be followed up during their treatment, in addition, the lost to follow up of patients are mainly young men and farmers^32^, Tola Habteyes Hailu's research results about the developing countries show that^33^ the main factors associated with lost to follow up and don't adherence to treatment are socioeconomic factors, including lack of transport costs, lack of social support and poor communication between patients and health care workers. Our study found that Buyi, Dong and Tujia have lower risk, while pure tuberculous pleurisy, severe and re-treatment have a higher risk (Table 2).

Age, household registration, occupation, source of patients, sputum smear results, and treatment type are the risk factors of treatment failed^34^, and the elderly, male, non-local, re-treatment, irregular treatment, delayed treatment and smoking will result in higher probability of Treatment failed^26,35^ (Table 3).

Age, gender, household registration, sputum smear results and treatment type are the risk factors of TB death^34^, while male, elderly, HIV-positive, etiological positive and re-treatment have a higher risk of death during anti-tuberculosis treatment^36^. Our study found that Dong, Tujia, retired persons and cadres and staffs have a lower risk, while students, middle-aged and elderly, referral, severe and re-treatment patients have a higher risk (Table 4).

Some studies have shown that TB patients with re-treatment and over 40 years old are more likely to develop MDR^37^. Our study shows that Miao and Buyi have a lower risk, while retired persons and cadres and staffs, middle-aged, referral, severe and retreated patients have a higher risk (Table 5).

## Conclusions

Some important findings and conclusions are drawn from our study. The variation trend of entirely adverse outcomes of TB in Guizhou province is becoming increasingly serious. Spatial clusters exists in each of the five adverse outcomes, among which some regions were not with high incidence of TB, which should be noticed by relevant departments. There are significant differences among nationalities in the adverse outcomes of TB, which may be greatly related to the differences in life styles, living habits, living environment and physical conditions among different nationalities, and it is also impossible to exclude the influence of some unclear genes among different nationalities. In the process of patient diagnosis and treatment, timely monitoring and active intervention should be carried out for high-risk groups, including middle-aged and elderly patients, rural patients, floating patients, severe patients and retreated patients, which is of great significance for reducing the rate of adverse reactions, lost to follow-up, died and switch to MDR. focus on strengthening the whole-course treatment management and health education for these high-risk groups, which is very important to improve patients' treatment compliance, completing follow-up, completing treatment and curing.

## Data Availability

The data used in this study are freely available. All data generated and analysed during the course of this study are available from the corresponding author upon request.

## References

[1] World Health Organization (2015). Global tuberculosis Report 2015.

[2] Zhang Y, Wang XL, Feng T, Fang CZ (2018). Analysis of spatial-temporal distribution and risk factors of pulmonary tuberculosis in China, during 2008–2015. Epidemiol. Infect..

[3] World Health Organization (2019). Global tuberculosis Report 2018.

[4] Zhou J, Chen HJ, Hong F, Li JL (2020). Analysis on the characteristics of tuberculosis patients in Guizhou Province from 2013 to 2018. Mod. Prev. Med..

[5] Liu Y, Xu CH, Wang ZY, Wang XM, Wang YH, Zhang H (2019). Investigation on the financial burden of tuberculosis patients in designated tuberculosis hospitals in China. Chin. J. Epidemiol..

[6] Li WB, Li XX, Zhang H, Li RZ, Cheng J, Wang LX (2012). Investigation on the status quo of medical security for non-drug-resistant tuberculosis patients in different regions of China. Chin. J. Prev. Med..

[7] Li CY, Cai XF, Yuan M (2019). Analysis of mental health status and risk factors of pulmonary tuberculosis patients treated in our hospital in recent three years. Chin. Health Stat..

[8] Fu T, Huang LJ, Yang JJ, Yao Z, Xiao H (2020). Analysis of 308 cases of MDR-TB patients' mental health status and its risk factors. Chin. J. Defense Consum..

[9] Qiang M, Cheng HZ, Da CZ, Ya HY (2019). Analysis on spatial-temporal distribution characteristics of smear positive pulmonary tuberculosis in China, 2004–2015. Int. J. Infect. Dis..

[10] Agarwal S, Nguyen DT, Teeter LD, Graviss EA (2017). Spatial-temporal distribution of genotyped tuberculosis cases in a county with active transmission. BMC Infect. Dis..

[11] Liu XF, Gou FX, Ren XW, Liu DP, Zheng YH, Wei KF (2015). Spatial-temporal specific incidence of pulmonary tuberculosis in Gansu, 2009–2013. Chin. J. Epidemiol..

[12] Silva MDAE (2016). Spatial distribution of tuberculosis from 2002 to 2012 in a midsize city in Brazil. BMC Public Health.

[13] Pfeiffer DU, Robinson TP, Stevenson M, Stevens KB, Clements ACA (2008). Spatial Analysis in Epidemiology.

[14] Li L, Xi Y, Ren F (2016). Spatio-temporal distribution characteristics and trajectory similarity analysis of tuberculosis in Beijing, China. Int. J. Environ. Res. Public Health.

[15] Jafari-Koshki T, Arsang-Jang S, Raei M (2015). Applying spatiotemporal models to study risk of smear-positive tuberculosis in Iran, 2001–2012. Int. J. Tuberc. Lung Dis..

[16] Kulldorff M, Nagarwalla N (1995). Spatial disease clusters: detection and inference. Stat. Med..

[17] Li XZ, Wang JF (2015). A fast method for making candidate clusters in spatial scan statistic method. ISPRS International Journal of Geo-Information. 2013; 15:505–511. Pulmonary Tuberculosis in the 7 Socioeconomic Regions and the 32 States of Mexico, 2000–2009. Arch. Bronconeumol. (English Edition).

[18] Anuj M, Pradeep D, Ajay D (2018). Determinants of adverse treatment outcomes among patients treated under Revised National Tuberculosis Control Program in Wardha, India: case–control study. Med. J. Armed Forces India.

[19] Deshmukh PR, Mundra A, Dawale A (2017). Social capital and adverse treatment outcomes of tuberculosis: a case-control study. Int. J. Tuberc. Lung Dis. Off. J. Int. Union Against Tuberc. Lung Dis..

[20] Anuj M, Pradeep RD, Ajay D (2017). Magnitude and determinants of adverse treatment outcomes among tuberculosis patients registered under Revised National Tuberculosis Control Program in a Tuberculosis Unit, Wardha, Central India: a record-based cohort study. J. Epidemiol. Glob. Health.

[21] Babalik A, Kiziltas S, Gencer S, Kilicaslan Z (2014). An investigation into the relationship between region specific quality of life and adverse tuberculosis treatment outcomes in Istanbul Turkey. Rev. Portuguesa Pneumol..

[22] Kassa GM, Teferra AS, Wolde HF, Muluneh AG, Merid MW (2019). Incidence andpredictors of lost to follow-up among drug-resistant tuberculosis patients at University of Gondar Comprehensive Specialized Hospital, Northwest Ethiopia: a retrospective follow-up study. BMC Infect. Dis..

[23] Leslie AE, Elizabeth DL, Tonya AM, Jessica E, Cynthia C, Botshelo K (2019). Investigating outcomes of adolescents and young adults (10–24 years of age) lost to follow-up from tuberculosis treatment in Gaborone, Botswana. Pediatr. Infect. Dis. J..

[24] Bemba ELP, Bopaka RG, Ossibi-Ibara R, Toungou SN, Ossale-Abacka BK, Okemba-Okombi FH (2017). Predictive factors of lost to follow-up status during tuberculosis treatment in Brazzaville. Rev. Pneumol. Clin..

[25] Matsumoto K, Komukai J, Kasai S, Hirota S, Koda S, Terakawa K, Shimouchi A (2014). Evaluation of risk factors for failed/defaulted on treatment outcomes of pulmonary tuberculosis in Osaka City. Kekkaku [Tuberculosis].

[26] Sharareh R, Niakan K, Mahshid N, Xiao JZ (2010). A logistic regression model to predict high risk patients to fail in tuberculosis treatment course completion. IAENG Int. J. Appl. Math..

[27] Juan JS-B (2015). Mortality Trends and Risk of Dying From Pulmonary Tuberculosis in the 7 Socioeconomic Regions and the 32 States of Mexico, 2000–2009. Arch. Bronconeumol. (English Edition).

[28] World Health Organization. Companion handbook to the WHO guidelines for the programmatic management of drug-resistant tuberculosis. Report on Geneva: 2014, 17–20. 25320836

[29] Jiang BF, Ma CC, Chen YG, Ba Y, Liu MD, Aliye A (2017). Analysis of adverse reaction rate and its risk factors of anti-tuberculosis drugs. Chin. J. Dis. Control.

[30] Ma Y, Xie ZY, Zang LN, Du J, Liu YH, Li L (2016). Analysis of factors risk adverse drug reactions in smear-positive pulmonary tuberculosis patients. Chin. J. Zoonoses.

[31] Aurora H, Kapoor S (2016). Determinants of lost to follow up during treatment among tuberculosis patients in Delhi. Int. J. Med. Res. Health Sci..

[32] Wu TY, Wei SS, Liu FY, Cao YF, Gao F (2013). Characteristics analysis of new smear positive pulmonary tuberculosis patients registered as missing in Guangxifrom 2008 to 2011. Chin. J. Dis. Control.

[33] Tola HH, Tol A, Shojaeizadeh D, Garmaroudi G (2015). Tuberculosis treatment non-adherence and lost to follow up among TB patients with or without HIV in developing countries: a systematic review. Iran. J. Public Health.

[34] Yang D, Wen J, Liu NQ, Ayinuer M, Qimanguli S, Hu X (2019). Factors affecting treatment outcomes of pulmonary tuberculosis in Xinjiang uygur population (article). Chin. Gen. Pract..

[35] Huang JY, Zhong Q, Zhou L, Chen L, Chen WQ (2014). Meta-analysis of factorsrisk tuberculosis treatment failure in China. Chin. J. Dis. Control.

[36] Xie Y, Han J, Yu WL, Li J, Sun X (2020). Analysis of the effect of short-range supervision on anti-tuberculosis treatment and the risk factors of death in patients with tuberculosis in Tianjin. Public Health China.

[37] Wang JJ, Zhou ML, Du YX, Chen C, Chen Z, Chen J (2017). Analysis of risk factors of drug-resistant/MDR-TB in Wuhan city. Chin. J. Dis. Control.

